# Association Between Respiratory Vaccines and Risk of Cognitive Impairment, Dementia and Alzheimer’s Disease: A Scoping Review

**DOI:** 10.3390/vaccines14090726

**Published:** 2026-08-22

**Authors:** Fernando M. Runzer-Colmenares, Nelson Luis Cahuapaza-Gutierrez, Cielo Cinthya Calderon-Hernandez, Camila Marjory Hilares-Jorge

**Affiliations:** 1 CHANGE Research Working Group, Universidad Científica del Sur, Lima 15067, Peru; 100065659@cientifica.edu.pe (N.L.C.-G.);; 2 Research Department, N y C—Center of Research and Medical Excellence (CRME), Lima 15067, Peru; 3 Faculty of Health Sciences, Psychology School, Universidad Científica del Sur, Lima 15067, Peru

**Keywords:** respiratory vaccines, cognitive impairment, dementia, Alzheimer’s disease, influenza vaccine, pneumococcal vaccine, COVID-19 vaccine, scoping review (source: MeSH NLM)

## Abstract

**Background/Objectives**: Neurocognitive disorders represent a growing burden among older adults. Although respiratory vaccines have demonstrated efficacy and safety in preventing acute respiratory events, their potential impact on the development of chronic neurocognitive disorders, such as cognitive impairment, dementia, and Alzheimer’s disease (AD), has not been extensively explored. The objective of this scoping review was to synthesize and analyze the available evidence on the association between respiratory vaccination and the risk of developing cognitive impairment, dementia, and Alzheimer’s disease. **Methods**: A scoping review was conducted in accordance with the PRISMA-ScR guidelines. A literature search was performed in the PubMed, Scopus, and Web of Science databases through 15 June 2026. Studies involving older adults (≥60 years), irrespective of their baseline neurocognitive status, were included. Observational studies (cohort and case–control studies) were considered. Editorials, narrative reviews, and other non-original articles were excluded. **Results**: Eighteen studies were included, with cohort studies being the predominant design. Four main categories of respiratory vaccines were evaluated: influenza, COVID-19, respiratory syncytial virus (RSV), and pneumococcal vaccines, in relation to the risk of cognitive impairment, dementia, and AD. Influenza vaccination was associated with a lower risk of dementia and AD, with a potential dose–response relationship observed, whereby a greater number of vaccinations was associated with a greater reduction in risk. Pneumococcal vaccination was also associated with a lower risk of dementia and AD, particularly among individuals who received a greater number of vaccine doses. In contrast, the evidence regarding COVID-19 and RSV vaccines was limited and yielded heterogeneous findings. **Conclusions**: Influenza and pneumococcal vaccination maybe associated with a lower risk of Alzheimer’s disease and dementia. However, the evidence regarding COVID-19 and RSV vaccines remains limited, and additional studies are needed to clarify their impact on the risk of developing neurocognitive disorders.

## 1. Introduction

Neurocognitive disorders (NCD) comprise a group of conditions characterized by the progressive decline of multiple cognitive domains, including memory, language, attention, executive functions, perceptual-motor function, and social cognition, with a substantial impact on autonomy and quality of life [1]. According to current diagnostic classifications, NCD are categorized into three main groups: delirium, mild neurocognitive disorder, and major neurocognitive disorder [2]. Their etiology is multifactorial and predominantly includes neurodegenerative diseases such as Alzheimer’s disease, Lewy body disease, and frontotemporal degeneration, as well as secondary causes including traumatic brain injury, infections, substance use, and alcohol abuse [2,3].

Common infections, particularly those severe enough to require hospitalization, including sepsis and pneumonia, have been consistently associated with an increased long-term risk of dementia. This effect persists even years after the acute event and after adjustment for pre-existing comorbidities [4,5]. In this context, vaccination represents one of the most effective public health interventions for preventing infectious diseases, particularly respiratory illnesses such as influenza, respiratory syncytial virus (RSV) infection, pneumonia, and COVID-19 [6]. Its implementation has contributed significantly to reducing morbidity and mortality worldwide, with particular benefits observed in vulnerable populations, including children, older adults, and individuals with comorbidities [7,8,9]. As vaccine coverage has expanded and new vaccine platforms have been developed, there has been growing interest in comprehensively evaluating their long-term safety profiles [10,11]. However, despite vaccines being an essential tool for preventing infectious diseases, their potential association with neurological adverse events has raised concerns among the general population and the scientific community [12]. Although vaccines are available for a wide range of infectious diseases, respiratory vaccines are of particular interest because they prevent infections that have been consistently associated with an increased long-term risk of cognitive decline and dementia. Infections such as influenza, pneumococcal disease, COVID-19, and RSV infection disproportionately affect older adults, are major causes of hospitalization and systemic inflammation, and may promote neuroinflammatory processes involved in the pathophysiology of neurocognitive disorders.

In this context, recent studies have explored potential associations between vaccination and neurological disorders, including Guillain–Barré syndrome (GBS), encephalitis, and neurodegenerative disorders, although most investigations have not found conclusive evidence supporting a direct causal relationship [13,14]. Nevertheless, public perception of risk may negatively influence vaccine acceptance, highlighting the importance of clear communication based on scientific evidence [15,16]. Within this framework, the relationship between respiratory vaccines and the risk of NCD has become an increasingly relevant topic in medical research, particularly in the context of widespread vaccination against diseases such as influenza and COVID-19. Regarding influenza vaccines, a protective effect against certain neurodegenerative disorders, such as Alzheimer’s disease, has been observed, particularly among older adults and individuals with chronic comorbidities [13,17]. However, some studies have reported an increased risk of demyelinating disorders in specific contexts, such as the use of pandemic vaccines in previous decades [13]. Conversely, COVID-19 vaccines have been scrutinized due to reports of rare neurological events, including GBS and Bell’s palsy, although these risks are significantly lower than the neurological complications associated with natural SARS-CoV-2 infection [18,19].

Despite challenges in interpreting available data, current evidence suggests that the benefits of respiratory vaccines substantially outweigh the potential risks of neurological adverse events. Continued research is essential to better understand the underlying mechanisms and develop strategies to minimize risks, particularly among vulnerable populations [13,20,21]. This scenario highlights the need for systematic reviews that integrate available evidence to inform data-driven vaccination policies and address public concerns. Within this framework, the primary objective of the present scoping review is to examine, synthesize, and analyze the current available evidence regarding the association between respiratory vaccines and the risk of developing cognitive impairment, dementia, and Alzheimer’s disease.

## 2. Materials and Methods

### 2.1. Protocol and Registration

The study protocol was developed according to the Preferred Reporting Items for Systematic Reviews and Meta-Analyses Protocols (PRISMA-P) 2015 guidelines. The review protocol was registered on the Open Science Framework (OSF) platform (https://osf.io/h2b7w/files/n52r8, accessed on 4 April 2026). This study was conducted and reported in accordance with the Preferred Reporting Items for Systematic Reviews and Meta-Analyses extension for Scoping Reviews (PRISMA-ScR) [22].

### 2.2. Eligibility Criteria

Eligibility criteria were based on the PICO research question previously established in the study protocol. The PICO framework was formulated according to its key components: P: population; I: intervention; C: comparator; O: outcomes.

#### 2.2.1. Inclusion Criteria

The population included older adults (≥60 years), irrespective of their baseline neurocognitive status. Studies were eligible if they included both cognitively healthy individuals and participants with previously diagnosed neurocognitive disorders (e.g., cognitive impairment, dementia, or Alzheimer’s disease). Baseline neurocognitive status was determined according to the definitions and diagnostic methods used in each primary study, including clinical assessment, International Classification of Diseases codes, validated cognitive assessment tools, or other diagnostic criteria reported by the study authors. The interventions included different respiratory vaccines, primarily influenza, RSV, pneumococcal, and COVID-19 vaccines, regardless of the number of doses (first, second, third, or booster dose) or vaccine platform. No specific comparator was required. Eligible outcomes included the incidence of neurocognitive disorders among participants without the disease at baseline, disease progression or other clinical outcomes among participants with pre-existing neurocognitive disorders, as well as the association between respiratory vaccination and the risk of these outcomes, as reported in the primary studies. Randomized controlled trials and observational studies (cohort and case–control studies) were included.

#### 2.2.2. Exclusion Criteria

Studies involving individuals younger than 60 years with chronic neurodegenerative diseases and/or neurological disorders, such as multiple sclerosis, amyotrophic lateral sclerosis, hereditary or acquired ataxias, muscular dystrophies, and neurocutaneous syndromes, among others, were excluded. Interventions other than respiratory vaccines were also excluded, including vaccines against herpes zoster, diphtheria, tetanus, pertussis, and poliomyelitis, among others. Additionally, studies consisting of letters to the editor, editorials, clinical images, comments, correspondence, brief communications, short reports, conference abstracts, literature reviews, narrative reviews, systematic reviews, meta-analyses, case reports and case series, quasi-experimental studies, preclinical studies, book chapters, books, and journalistic or opinion articles were excluded.

### 2.3. Information Sources

The preliminary search was conducted on 1 January 2026, and updated on 5 March 2026. The final search reported in this study was performed on 15 June 2026. A systematic literature search was conducted in the following electronic databases: PubMed and Scopus, as well as the Web of Science platform. To maximize comprehensiveness, the reference lists of the included studies were manually screened.

### 2.4. Search Strategy

At this stage, a search strategy was developed using controlled vocabulary terms from the Medical Subject Headings (MeSH) database of the National Library of Medicine (NLM), including: Vaccines, Influenza Vaccines, Respiratory Syncytial Virus Vaccines, Pneumococcal Vaccines, and COVID-19 Vaccines. These terms were combined with the terms Cognitive Impairment, Dementia, and Alzheimer’s Disease using Boolean operators (AND, OR). No publication date restrictions were applied; however, the search was limited to studies published in English. The detailed search strategies for each database are provided in Supplementary Material S1.

### 2.5. Study Selection Process

All references were imported into EndNote X9 for duplicate removal. Subsequently, records were exported to the Rayyan QCRI web platform (https://www.rayyan.ai/ (accessed on 25 June 2026)). Two authors (NLCG and CCCH) independently screened potentially eligible studies by reviewing titles and abstracts. In the second stage, the selected articles were assessed in full text to determine eligibility. Any disagreements were resolved through consensus between reviewers and, when necessary, a third author (FMRC) was consulted.

### 2.6. Data Extraction Process

The most relevant characteristics of the included studies were extracted, including first author, year of publication, country, study design, follow-up period, inclusion and exclusion criteria, total sample size, type of neurocognitive disorder, vaccinated population, vaccine type, number of doses, age, sex, control group, outcomes, and conclusions. Any discrepancies were resolved through consensus among the authors.

### 2.7. Data Synthesis

Given the nature of the study design (scoping review), no formal assessments of risk of bias, methodological quality, or certainty of evidence were performed. Likewise, no meta-analysis or advanced statistical modeling was conducted. Instead, a qualitative synthesis was performed, complemented by descriptive and basic analytical analyses. All analyses were conducted using STATA software, version Now19 SE.

## 3. Results

### 3.1. Selection of Evidence Sources

**Figure 1:**
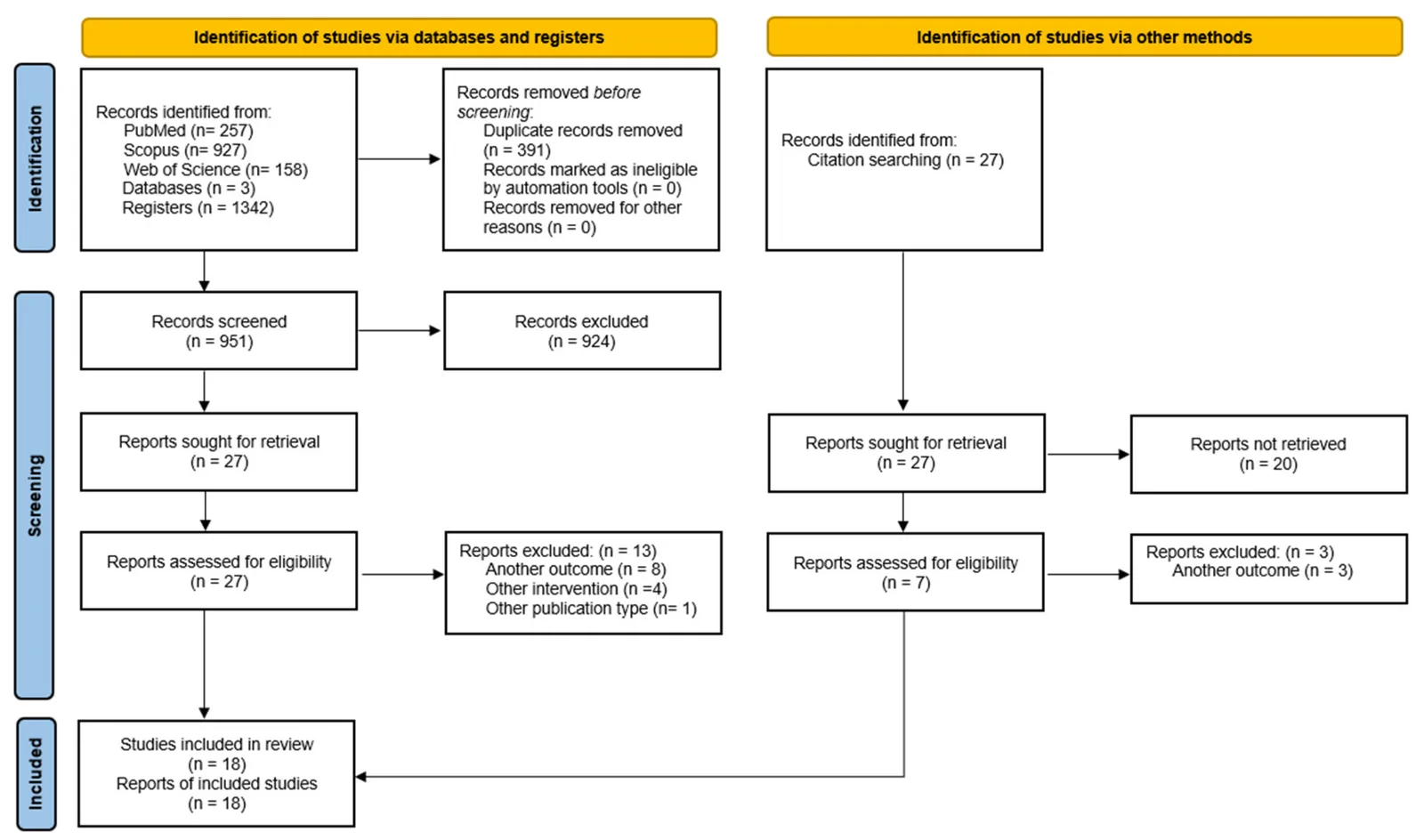
Study selection process for respiratory vaccines and neurocognitive disorders. PRISMA 2020 flow diagram.

A comprehensive search was conducted across three databases (PubMed, Scopus, and Web of Science). Subsequently, 391 records were removed as duplicates. During title and abstract screening, a total of 924 manuscripts were excluded. After full-text assessment, 13 records were excluded due to inappropriate outcomes, interventions or publication type. Additionally, the reference lists of 27 studies were manually reviewed, leading to the identification of 4 additional manuscripts. Finally, the present scoping review included a total of 18 studies. Figure 1 provides a detailed overview of the study selection process.

### 3.2. Characteristics of the Included Studies

The association between respiratory vaccines and the risk of neurocognitive disorders was evaluated in 18 observational studies, of which 10 assessed influenza vaccines, 2 assessed COVID-19 vaccines, 1 assessed respiratory syncytial virus vaccine, and 5 assessed pneumococcal vaccines. The main neurocognitive outcomes evaluated were cognitive impairment, AD, and dementia.

In most included studies, the evaluated populations had a mean age above 60 years. The specific findings for each respiratory vaccine and its association with the risk of neurocognitive disorders are described below. Detailed characteristics of the included studies are summarized in Table 1.

### 3.3. Influenza Vaccines

Ten observational studies were included, of which nine were retrospective cohort studies and one was a case–control study. The studies were published between 2001 and 2026, with follow-up periods spanning 1991 to 2022. Geographically, most studies were conducted in North America (*n* = 5), including the United States and Canada, followed by Asia (*n* = 3), with studies conducted in Taiwan and China, and Europe (*n* = 2), represented by Denmark and the United Kingdom. Collectively, the studies evaluated 2,958,984 vaccinated individuals, with a mean age predominantly over 60 years. The included studies primarily assessed the association between influenza vaccination and the risk of developing Alzheimer’s disease and dementia (Table 1).

Tyas et al. [23] conducted a population-based longitudinal study to analyze various risk factors associated with the development of AD. After five years of follow-up, influenza vaccination was identified as an associated factor, and analyses showed a reduced risk of developing AD. Consistently, Verreault et al. [24] evaluated the association between prior exposure to conventional vaccines (diphtheria/tetanus, poliomyelitis, and influenza vaccines) and the risk of AD. Their findings indicated that influenza vaccination, as well as the other evaluated vaccines, was associated with a lower risk of developing AD. Similarly, Bukhbinder et al. [28], through a comparison between individuals with and without previous influenza vaccination, also reported that influenza vaccination was associated with a reduced risk of AD.

Liu et al. [25] estimated the relationship between influenza vaccination and dementia risk among patients with chronic kidney disease, and found a lower risk of dementia in vaccinated individuals compared with unvaccinated individuals. Similarly, Luo et al. [26] investigated the association between influenza vaccination among patients with chronic obstructive pulmonary disease, finding that vaccinated individuals had a lower risk of dementia. They also observed a dose–response relationship, in which a greater number of vaccinations was associated with a greater reduction in risk.

Wiemken et al. [27], through a retrospective cohort study, analyzed the cumulative effect of influenza vaccination on dementia risk. Their results showed that vaccinated individuals had a lower risk of developing dementia compared with unvaccinated individuals. Although individuals receiving 1, 2, or 3–5 vaccinations showed similar risks compared with the unvaccinated group, administration of ≥6 influenza vaccine doses was associated with a significant reduction in dementia risk. Other studies evaluated specific vaccination characteristics and their relationship with neurocognitive outcomes. Zhao et al. [31] observed that influenza vaccination was associated with a dose–response reduction in dementia risk. Conversely, Bukhbinder et al. [32] compared responses between standard-dose and high-dose influenza vaccines among adults aged ≥65 years, finding that the high-dose formulation was associated with a lower risk of AD, with a more pronounced effect among women. Furthermore, Lophatananon et al. [29] investigated the association between herpes zoster and influenza vaccination and the risk of developing dementia. Their results showed that influenza vaccination was also associated with a slight reduction in dementia risk. Finally, not all studies reported consistent findings. Appel et al. [30], using a cohort design, found that influenza vaccination during follow-up was associated with slightly higher risk of dementia, after adjustment for sociodemographic factors and comorbidities, both within and beyond the first five years after the initial vaccination. However, they observed that the dementia hazard rate decreased as the number of vaccinations increased, with the reduction being more evident among individuals receiving six or more doses.

### 3.4. COVID-19 Vaccines

Two observational studies were included: one retrospective cohort study and one nested case–control study. Both studies were published between 2024 and 2026, with follow-up periods spanning 2020 to 2022. The geographic distribution analysis showed that both studies were conducted in Asia, specifically one in the Republic of Korea and the other in Israel. Collectively, the studies evaluated 534,891 vaccinated individuals. The mean age of the study populations was over 60 years, and the effects of COVID-19 vaccination were evaluated in relation to various neurocognitive disorders, including cognitive impairment, dementia, and AD. mRNA-based vaccines, primarily BNT162b2 (Pfizer–BioNTech) and mRNA-1273 (Moderna), were the most extensively evaluated. Detailed characteristics of the included studies are presented in Table 1.

The retrospective cohort study by Roh et al. [33] investigated the association between COVID-19 vaccination and the risk of developing of AD and its prodromal stage, mild cognitive impairment (MCI). After a three-month follow-up period, the authors observed that individuals vaccinated with messenger RNA (mRNA)-based vaccines had a higher risk of AD and MCI compared with the unvaccinated group. Similarly, Zur et al. [34], using a nested case–control design, evaluated the risk of developing dementia among previously healthy individuals exposed to mRNA-based COVID-19 vaccination. The results showed that vaccination was associated with a 7%, 15%, and 31% lower risk of dementia after receiving two, three, and four doses, respectively.

### 3.5. Respiratory Syncytial Virus Vaccines

A single study was identified that evaluated the effect of respiratory syncytial virus vaccines on dementia risk. Taquet et al. [35] analyzed propensity score-matched cohorts including 436,788 individuals and observed that herpes zoster vaccination and adjuvanted RSV vaccine containing AS01, either individually or in combination, were associated with a lower risk of dementia during an 18-month follow-up period. However, because the available evidence is limited to a single study in which the adjuvanted RSV vaccine was evaluated together with herpes zoster vaccination. Therefore, these results should be interpreted with caution and require confirmation through future studies specifically designed to evaluate the independent effect of RSV vaccination on dementia risk in larger populations using prospective study designs.

### 3.6. Pneumococcal Vaccines

Five observational studies were included, of which four were cohort studies (retrospective and prospective designs) and one was a case–control study. Publication years ranged from 2021 to 2024, while follow-up periods ranged from 1970 to 2022. The continental distribution analysis showed that most studies were conducted in North America (United States), followed by Europe (United Kingdom) and Asia (Japan). Collectively, the included studies evaluated 304,212 vaccinated individuals. The mean age of the study populations was predominantly above 70 years. The included studies primarily assessed the association between pneumococcal vaccination and the risk of developing AD and dementia.

Harris et al. [37] analyzed, through a retrospective cohort study, the risk of developing AD among adults with and without previous vaccination against tetanus/diphtheria, herpes zoster, or pneumococcus. The authors evaluated each vaccine separately rather than the combined vaccination status. Among individuals receiving pneumococcal vaccination, 7.92% (*n* = 20,583) developed AD during follow-up, compared with 10.9% (*n* = 28,558) of unvaccinated individuals. These findings suggested that pneumococcal vaccination was associated with a lower risk of developing AD. Similarly, Huo et al. [39], through a matched cohort analysis, evaluated the association between pneumococcal vaccination and AD risk. The results showed that vaccinated individuals had a significantly lower risk of developing AD compared with those without pneumococcal vaccination.

Other studies explored the effect of pneumococcal vaccination considering individual characteristics and genetic factors. Ukraintseva et al. [38], using a retrospective cohort design, estimated the association between receipt of pneumococcal polysaccharide and influenza vaccines between ages 65 and 75 years and the development of AD after age 75 years, considering the NECTIN2 gene rs6859 polymorphism as a risk factor. Their results showed that a greater cumulative number of pneumonia and influenza vaccinations was associated with lower odds of AD in the overall sample and among carriers of the A allele, although this association was not observed among non-carriers. Finally, Iwai-Saito et al. [40], through longitudinal analyses, evaluated the association between pneumococcal vaccination and dementia risk. Their findings indicated that vaccination with the 23-valent pneumococcal polysaccharide vaccine (PPSV23) was inversely associated with dementia incidence during both short- and long-term follow-up periods. However, Wilkinson et al. [36], through a population-based cohort study evaluating various medications, including pneumonia vaccines, observed that vaccination was associated with a high risk of dementia (Table 1).

## 4. Discussion

The main findings of this scoping review suggest that influenza and pneumococcal vaccination may be associated with a lower risk of Alzheimer’s disease and dementia. However, the evidence regarding COVID-19 and respiratory syncytial virus (RSV) vaccination remains limited.

Regarding influenza vaccines, most included studies reported an association with a lower risk of developing AD and dementia, with patterns that further support the biological plausibility of this association. In particular, the dose–response relationship observed in several studies is noteworthy, as a greater cumulative number of vaccinations was associated with a greater reduction in risk, with administration of ≥6 doses being the threshold most frequently associated with a significant benefit. These findings are consistent with previous studies in which influenza vaccination was associated with a dose-dependent reduction in the risk of developing dementia [17,41]. An important finding of this review was the apparent dose–response relationship observed for influenza vaccination, whereby repeated annual vaccination was associated with a greater reduction in the risk of neurocognitive disorders than a single vaccination. This observation has also been reported in recent systematic reviews and meta-analyses, which consistently demonstrated a greater reduction in risk among individuals who received multiple doses of the influenza vaccine over time [17]. One biologically plausible explanation is that repeated vaccination reduces the cumulative burden of influenza and its related complications, thereby limiting recurrent episodes of systemic inflammation, a process that has been implicated in neuroinflammatory pathways associated with cognitive decline. This hypothesis is particularly relevant because influenza is highly prevalent and disproportionately affects older adults, whose age-related immunosenescence increases both their susceptibility to infection and the severity of its clinical consequences. Nevertheless, direct mechanistic evidence supporting this hypothesis remains limited. Alternatively, the observed association may be partially explained by the “healthy vaccinee effect”, whereby individuals who receive annual influenza vaccination are more likely to engage in preventive health behaviors, adhere to medical recommendations, and have better access to healthcare services, all of which may independently contribute to a lower risk of neurocognitive disorders. Therefore, although the observed dose–response relationship is biologically plausible, it should be interpreted with caution until future prospective and mechanistic studies clarify the extent to which it reflects a true biological effect rather than residual confounding.

Regarding COVID-19 vaccines, the findings were substantially less consistent. While Zur et al. [34] reported a reduced risk of dementia and observed a potential dose–response relationship, Roh et al. [33] documented an increased risk of AD and mild cognitive impairment following vaccination with messenger RNA (mRNA)-based vaccines. This heterogeneity may be explained by differences in follow-up duration, study design, evaluated populations, and vaccine platforms analyzed, limiting the ability to draw definitive conclusions regarding this specific association. Regarding pneumococcal vaccines, most studies reported a potential reduction in the risk of AD among adults aged ≥65 years. The findings also suggest a possible interaction with genetic factors and underlying immunogenetic mechanisms that have not yet been fully elucidated. Finally, although the available evidence on RSV vaccination also suggests a potential reduction in risk, it is based on a single study and should therefore be interpreted as a preliminary finding that requires confirmation through further research.

The plausible biological mechanisms underlying the association between vaccination and reduced dementia risk remain incompletely understood. One possible explanation is that vaccination against respiratory viruses reduces the likelihood of developing respiratory infections and other vaccine-preventable pulmonary diseases. Several studies have demonstrated that infectious events can impair cognitive function and increase the risk of dementia. In this context, respiratory vaccines may reduce the risk of neurocognitive disorders by preventing infections and, consequently, decreasing virus-induced systemic inflammation and neuroinflammation [42]. Additionally, vaccines may exert immunomodulatory effects through immune training, promoting more efficient innate immune responses, including the activation of physical barriers, immune cells, and immune proteins capable of contributing to the clearance of pathological proteins, such as beta-amyloid plaques, which are involved in the pathophysiology of Alzheimer’s disease [41]. Furthermore, infections have been described as potential contributors to an increased risk of vascular events, such as myocardial infarction and stroke, which are relevant factors in the development of dementia [43]. In this regard, recent studies have evaluated the impact of respiratory vaccines among older adults with cardiovascular diseases, showing reductions in hospitalizations, adverse cardiovascular events, and mortality [44]. This may represent shared mechanisms and pathways underlying both cardioprotective and neuroprotective effects.

Additionally, recent research has investigated the potential mechanisms through which herpes zoster vaccination may reduce dementia risk [45]. It has been proposed that AS01-adjuvanted vaccines may support the trained immunity hypothesis as a biological pathway underlying the association between vaccination and lower neurocognitive risk [45]. This process may involve a feedback loop between innate and adaptive immunity, in which interferon-gamma (IFN-γ) plays a central role. Vaccination with an AS01 adjuvant may initially induce antigen presentation by dendritic cells and monocytes, promoting CD4^+^ T-cell activation and, through the release of interleukin (IL)-12 and IL-18, the activation of natural killer (NK) cells [45]. This response may be further enhanced by IL-2 produced by CD4^+^ T cells themselves. Subsequently, both activated cell populations produce IFN-γ, whose signaling feeds back onto monocytes and induces epigenetic modifications that constitute the molecular basis of trained innate immunity [46]. However, it remains unclear whether these immunomodulatory effects are primarily driven by the AS01 adjuvant, the vaccine antigen, the vaccine platform, or the broader immune response elicited by vaccination. Moreover, most mechanistic evidence supporting the trained immunity hypothesis has been derived from studies of AS01-adjuvanted vaccines rather than respiratory vaccines specifically. Therefore, extrapolating these mechanisms to respiratory vaccines should be considered a biologically plausible but as yet unproven hypothesis. Additional mechanistic and clinical studies are needed to determine whether these pathways independently contribute to the potential association between respiratory virus vaccination and a lower risk of neurocognitive disorders.

Overall, this circuit may be interpreted as a mechanism whereby the initial activation of vaccine-induced innate immune responses subsequently stimulates adaptive immunity, which in turn epigenetically “reprograms” innate immune cells, providing the immune system with prolonged functional memory and a potential capacity to modulate systemic inflammation and neuroinflammation associated with neurodegenerative processes [46]. Similar immunomodulatory mechanisms may also be proposed for respiratory vaccines, opening new research avenues aimed at clarifying the mechanistic pathways through which these vaccines may contribute to reducing the risk of neurocognitive disorders.

Beyond the direct effects of respiratory vaccination, hypothetical biological mechanisms may also contribute to the observed associations with neurocognitive outcomes. The intestinal–vagal interoceptive pathway has been proposed as a potential link between peripheral immune responses and brain function, through modulation of systemic inflammation and neuroimmune signaling. Under conditions of systemic immune activation, such as those associated with respiratory infections or vaccine-induced immune responses, this signaling dysregulation could promote neuroinflammation by enhancing the trafficking of inflammatory mediators, altering blood–brain barrier homeostasis, and compromising neural networks involved in memory and executive function [47,48]. However, this mechanism remains speculative and is not directly supported by the studies included in this review. Further mechanistic research is required to determine whether alterations in gut–brain communication contribute to the relationship between respiratory immune exposures and neurocognitive disorders.

### 4.1. Limitations

This study has several limitations. First, although an extensive literature search was conducted across multiple databases, the search was restricted to publications in English, which may have excluded relevant evidence published in other languages and introduced language selection bias. Second, the substantial methodological heterogeneity among the included studies, both in terms of study design (retrospective cohorts, prospective cohorts, and case–control studies), vaccine platforms evaluated, follow-up periods, and study populations, prevented the performance of a robust quantitative analysis capable of generating pooled effect estimates and statistically comparable results across different vaccine types. Third, by nature, scoping reviews are designed to prioritize the mapping and narrative synthesis of available evidence rather than quantitative evidence aggregation. Therefore, no formal meta-analysis was performed despite the heterogeneity observed among the included studies. Fourth, a particularly relevant limitation is that many studies included general older adult populations, in which individuals with a previous diagnosis of cognitive impairment, Alzheimer’s disease, or dementia represented only a subset of the total cohort, or alternatively excluded individuals with pre-existing neurocognitive diagnoses at baseline. Although we attempted to extract and analyze neurocognitive-specific outcomes whenever possible, many studies did not report stratified results according to baseline cognitive status or the presence of neurodegenerative risk factors. Consequently, some reported outcomes may not exclusively reflect the effect of vaccination among populations at risk or with specific neurocognitive vulnerability, which may limit the direct extrapolation of findings to this target population. Since the purpose of a scoping review is to map available evidence rather than generate pooled effect estimates, a more precise quantification of the potential benefit of vaccination among individuals with cognitive impairment or well-defined neurodegenerative risk will require studies with systematic subgroup reporting or meta-analyses specifically designed for this purpose. Fifth, the biological mechanisms underlying the potential association between vaccination and neurocognitive protection remain incompletely understood. In particular, evidence regarding intestinal interoceptive dysfunction and its potential role as a mediating pathway linking vaccination, systemic neuroinflammation, and the development of neurocognitive disorders within the broader gut–brain axis framework remains limited. Therefore, any mechanistic interpretation proposed in this review, including hypotheses related to neuroinflammation modulation or trained immunity, should be considered exploratory and hypothetical rather than established causal evidence. Further mechanistic studies, ideally incorporating simultaneous assessment of inflammatory biomarkers, the gut microbiome, and blood–brain barrier integrity, are required to clarify these pathways. Sixth, it is important to distinguish between evidence derived directly from the included studies and the biological mechanisms proposed to explain the observed associations. Although the included studies consistently reported that certain respiratory vaccines were associated with a lower risk of neurocognitive disorders, none were designed to investigate the biological mechanisms underlying these associations. Therefore, explanations involving infection prevention, reduced systemic inflammation and neuroinflammation, trained immunity, adjuvant-related immunomodulation, or differences between vaccine platforms should be regarded as biologically plausible hypotheses supported primarily by indirect experimental or mechanistic evidence. Further mechanistic studies and well-designed prospective investigations are required to determine whether these processes play a causal role in the observed association between vaccination and a lower risk of neurocognitive disorders. Finally, a considerable proportion of the included studies did not provide sufficient information regarding clinical and biological variables that may influence immune responses and neurocognitive risk, such as comorbidities, functional status, cognitive reserve, nutritional status, frailty, or immunosenescence status. The systematic absence of these covariates limits the ability to determine whether the observed protective effect results from a direct immunological mechanism of vaccination or instead reflects residual confounding associated with greater healthcare access, better functional status, or lower comorbidity burden among vaccinated individuals.

### 4.2. Perspectives

From a public health perspective, the potential association between respiratory vaccination and a lower risk of neurocognitive disorders is particularly relevant in aging populations, where increased susceptibility to infections, frailty, and a growing burden of dementia converge. Available evidence suggests that vaccines such as influenza and pneumococcal vaccines may provide benefits that extend beyond the prevention of acute disease, supporting their incorporation into healthy aging strategies and approaches aimed at preventing cognitive decline. Nevertheless, these findings should be interpreted cautiously, as the predominance of observational studies introduces the possibility of healthy participant bias or the healthy vaccinee effect, whereby vaccinated individuals may have more favorable baseline health profiles and preventive behaviors that influence effect estimates. Although the underlying mechanisms remain under investigation, modulation of systemic neuroinflammation and, indirectly, the involvement of the gut–brain axis and intestinal interoception represent priority areas for future research [45,48,49,50,51].

From a neuropsychological perspective, the findings of this study are particularly relevant because neurocognitive disorders involve not only progressive impairment of higher cognitive functions but also significant alterations in adaptive functioning, functional independence, emotional regulation, and overall well-being during aging. From this perspective, the potential association between respiratory vaccines and a lower incidence of cognitive decline, dementia, and Alzheimer’s disease may be interpreted as a complementary component of a preventive approach aimed at promoting healthy aging and preserving cognitive function. Furthermore, these findings reinforce the importance of promoting evidence-based preventive behaviors through strategies aimed at improving health psychoeducation, reducing the impact of misinformation and vaccine hesitancy, and thereby enhancing adherence to vaccination programs. In addition, the biopsychosocial model suggests that prevention of neurocognitive decline extends beyond the biomedical domain and also involves psychological and behavioral factors that influence the development of preventive behaviors and the psychosocial well-being of older adults.

### 4.3. Recommendations

Based on the findings of this scoping review, the design and implementation of large-scale randomized controlled trials are recommended to prospectively evaluate the effects of respiratory vaccines, particularly influenza and pneumococcal vaccines, given the potential consistency of the available observational evidence regarding the reduction in the risk of cognitive impairment, Alzheimer’s disease, and dementia. These studies should ensure adequate representation of advanced-age subgroups, individuals with genetic risk factors, and populations with concomitant neurological or cardiovascular comorbidities. Furthermore, it is essential that future studies standardize the assessment of neurocognitive outcomes using validated and harmonized instruments and incorporate biomarkers of neurodegeneration and immune response. This would facilitate the exploration of the biological mechanisms underlying the observed associations, including modulation of neuroinflammation, reduction in recurrent systemic infections and their indirect neurotoxic effects, and trained immunity-related phenomena. Finally, public health strategies should be implemented to improve the coverage, accessibility, and uptake of respiratory vaccination among older adults, together with the establishment of longitudinal surveillance systems capable of evaluating its effects under real-world clinical conditions.

## 5. Conclusions

Respiratory vaccines, particularly those targeting influenza and pneumococcal disease, may be associated with a lower risk of developing dementia and Alzheimer’s disease. In contrast, evidence regarding COVID-19 and respiratory syncytial virus vaccines remains limited and heterogeneous, preventing definitive conclusions. Since the available evidence is predominantly derived from observational studies, these findings should be interpreted with caution, as residual confounding and other sources of bias cannot be ruled out.

## Figures and Tables

**Table 1:** Characteristics of the included studies.

Author/Year	Study Design	Follow-Up Period	Inclusion Criteria	Exclusion Criteria	Total Sample Size	Vaccine Type	Vaccinated	Age	Female	Unvaccinated	Age	Female	Outcomes Neurocognitive Disorders	Conclusions
Tyas et al., 2001 (Canada) [23]	Case–control	1991–1997	≥65 years old	<65 years old	646	Influenza	31	79.8	24 (66.7)	615	73.7	409 (62.2)	RR: 0.40 (95% CI: 0.17–0.96); adjusted for age, sex and education	Influenza vaccination is associated with lower risk of AD
Verreault et al., 2001 (Canada) [24]	Retrospective cohort	1991–1992	≥65 years old	<65 years old with MCI/Dementia	3865	Influenza	2178	-	-	1687	-	-	OR: 0.75 (95% CI: 0.54–1.04); adjusted for age, sex and education	Influenza vaccination is associated with lower risk of AD
Liu et al., 2016 (Taiwan) [25]	Retrospective cohort	2000–2007	≥60 years old	<60 years old	11,943	Influenza	5745	73	2394 (41.67)	6198	72.81	2768 (44.66)	aHR: 0.68; adjusted for age, sex, comorbidities, level of urbanization and monthly income	Influenza vaccine is associated with a lower risk of dementia
Luo et al., 2020 (Taiwan) [26]	Retrospective cohort	2001–2012	≥60 years old	<60 years old	19,848	Influenza	10,980	72.06	4709 (42.89)	8868	71.76	3658 (41.25)	aHR: 0.68 (95% CI: 0.62–0.74); adjusted for age, sex, comorbidities, level of urbanization and monthly income	Influenza vaccination associated with a lower risk of dementia
Wiemken et al., 2021 (USA) [27]	Retrospective cohort	2011–2019	≥65 years old no dementia	<65 years old dementia Vaccination	123,747	Influenza	66,822	75.5	2512 (3.8)	56,925	75.5	2237 (3.9)	HR: 0.86 (95% CI: 0.83–0.88); adjusted for weighted	Influenza vaccination is associated with lower risk of dementia
Bukhbinder et al., 2022 (USA) [28]	Retrospective cohort	2009–2019	≥65 years old no dementia	<65 years old MCI/Dementia	1,871,774	Influenza	935,887	73.7	531,644 (56.8)	935,887	73.7	533,387 (57)	RR: 0.60 (95% CI: 0.59–0.61); not adjusted	Influenza vaccination associated with a lower risk of dementia
Lophatananon et al., 2023 (UK) [29]	Retrospective cohort	2013–2020	≥70 years old without dementia	<70 years old dementia	7,962,257	Influenza	742,487	-	398,027 (53.61)	7,219,770	NR	3,868,487 (53.58)	aHR: 0.71 (95% CI: 0.69–0.72); adjusted for age, gender, and practice-level factors	Influenza vaccination is associated with lower risk of dementia
Appel et al., 2024 (Denmark) [30]	Retrospective cohort	2002–2018	≥65 years old	<65 years old	1,673,421	Influenza	991,729	82.9	531,928 (53.6)	681,692	81.6	366,725 (53.8)	1 dose; IRR: 1.14 (95% CI: 1.12–1.17) ≥6 doses; IRR: 0.95 (95% CI 0.93–0.97); adjusted for sociodemographic factors and comorbidities	Influenza vaccination did not show a clear effect against dementia
Zhao et al., 2024 (China) [31]	Retrospective cohort	NR	≥60 years old	<60 years old	70,938	Influenza	38,328	64.0	20,257 (52.85)	32,610	64.9	17,915 (54.94)	HR: 0.83 (95% CI: 0.72–0.95); adjusted for comorbidities and medication use	Influenza vaccination is associated with lower risk of dementia
Bukhbinder et al., 2026 (USA) [32]	Retrospective cohort	2014–2019	≥65 years old	<65 years old	164,797	Influenza	HD: 120 775 SD: 44 022	HD: 74.4 SD: 73.0	HD: 106 156 (57.3) SD: 30 525 (56.4)	0	-	-	-	High doses of influenza vaccine are associated with a lower risk of AD
Roh et al., 2024 (Republic of Korea) [33]	Retrospective cohort	2020–2021	≥65 years old vaccinated	Not vaccinated	558,017	COVID-19	519,330	72.73	269,358 (51.8)	38,687	75.23	22,941 (59.3)	MCI; OR: 2.3 (95% CI: 1.84–3.06) AD; OR: 1.22 (95% CI: 1.02–1.46); not adjusted	The COVID-19 vaccine increased the risk of MCI and AD
Zur et al., 2026 (Israel) [34]	Nested case–control	2020–2022	≥60 years without dementia	Patients with dementia	173,293	COVID-19	15,561	78.2	-	157,732	77.5	-	OR: 0.89 (95% CI: 0.85–0.94); adjusted for socioeconomic status, ethnicity and comorbidities	COVID-19 vaccination reduces the risk of developing dementia
Taquet et al., 2025 (UK) [35]	Retrospective cohort	2023	≥60 years old	<60 years old	71,876	RSV	35,938	72.84	20,883 (58.11)	35,938	-	-	-	RSV vaccine is associated with a lower risk of dementia
Wilkinson et al., 2021 (UK) [36]	Case–control	1970–2018	≥60 years old Dementia	<60 years old	551,344	Pneumococcus	16,998	-	-	534,346	-	-	HR: 1.11; adjusted for sex, smoking and socioeconomic status	Pneumococcus vaccines were associated with a high risk of dementia
Harris et al., 2023 (USA) [37]	Retrospective cohort	2009–2019	≥65 years old AD	<65 years old	1651,991	Pneumococcus	270,835	72.2	156,696 (57.85)	270,835	72.2	156,702 (57.86)	RR: 0.73 (95% CI: 0.71–0.74); adjusted for weighted	Pneumococcal vaccination is associated with a lower risk of AD
Ukraintseva et al., 2023 (USA) [38]	Retrospective cohort	NR	≥65 years old	<65 years old AD	5599	Pneumococcus	1984	-	1182 (59.58)	3615	-	-	OR: 0.94 (95% CI: 0.89–0.99); adjusted for sex, race, birth cohort, education, and smoking	Pneumococcal vaccination is associated with lower risk of AD
Huo and Finkelstein 2024 (USA) [39]	Retrospective cohort	September 2013–August 2019	≥65 years old	<65 years old MCI/Dementia Influenza vaccine Medication for AD	157,269	Pneumococcus	14,395	74.7	7927 (55.1)	142,874	76.2	79,475 (55.6)	OR: 0.37 (95% CI: 0.33–0.42); adjusted for sex, age and comorbidities	Pneumococcal vaccination reduced the risk of AD
Iwai-Saito et al., 2024 (Japan) [40]	Prospective cohort	2013–2022	≥65 years old	<65 years old	9838	Pneumococcus	4220	72.97	5316 (53.9)	5618	72.94	NR	HR: 0.77 (95% CI: 0.63–0.95), short-term follow-up HR: 0.83 (95% CI: 0.70–0.97), long-term follow-up; adjusted for age, sex, educational years and other outcomes	Pneumococcal vaccination is negatively associated with dementia

AD: Alzheimer’s disease; aHR: adjusted hazard ratio; CI: confidence interval; HD: high dose; HR: hazard ratio; IRR: incidence rate ratios; MCI: mild cognitive impairment; ND: neurocognitive disorders; NR: not reported; OR: odds ratio; RR: risk ratio; RSV: respiratory syncytial virus; SD: standard dose.

## Data Availability

Additional data related to this paper may be requested from the authors.

## Supplementary Materials

The following supporting information can be downloaded at: https://www.mdpi.com/article/10.3390/vaccines14090726/s1, Supplementary Material S1, Design of Search Strategy.

## Abbreviations

The following abbreviations are used in this manuscript:

AD	Alzheimer’s disease
IFN-γ	Interferon-gamma
IL	Interleukin
MCI	Mild cognitive impairment
NCD	Neurocognitive disorders
RSV	Respiratory syncytial virus
