# Supplementary material for: Association Between Respiratory Vaccines and Risk of Cognitive Impairment, Dementia and Alzheimer’s Disease: A Scoping Review
Source: Vaccines (Basel). 2026 Aug 22;14(9):726. doi: 10.3390/vaccines14090726 (PMC13611525; doi:10.3390/vaccines14090726)
Supplement: Supplementary file 1 [file vaccines-14-00726-s001.zip › vaccines-4442124-supplementary.pdf]

Supplementary Material S1. Design of Search Strategy.

June 15, 2026

Data bases: PubMed, Scopus, EMBASE and Web of Science.

PubMed (n= 257)

#1: (Influenza Vaccines[MeSH] OR "Influenza Vaccine*"[TIAB] OR "Influenza Virus Vaccine*"[TIAB] OR "Universal Influenza Vaccine*"[TIAB] OR Respiratory Syncytial Virus Vaccines[MeSH] OR "Respiratory Syncytial Virus Vaccine*"[TIAB] OR "RSV Vaccine*"[TIAB] OR Pneumococcal Vaccines[MeSH] OR "Pneumococcal Vaccine*"[TIAB] OR "Pneumococcal Polysaccharide Vaccine*"[TIAB] OR Pneumovax[TIAB] OR COVID-19 Vaccines[MeSH] OR "COVID-19 Vaccine*"[TIAB] OR "SARS2 Vaccine*"[TIAB] OR "Coronavirus Disease 2019 Vaccine*"[TIAB] OR "2019-nCoV Vaccine*"[TIAB] OR "SARS-CoV-2 Vaccine*"[TIAB] OR "COVID-19 Virus Vaccine*"[TIAB] OR BNT162 Vaccine[MeSH] OR "BNT162 Vaccine*"[TIAB] OR "COVID-19 Vaccine Pfizer-BioNTech*"[TIAB] OR Tozinameran[TIAB] OR "BNT-162B2*"[TIAB] OR Comirnaty[MeSH] OR Comirnaty[TIAB] OR "Pfizer Covid-19 Vaccine*"[TIAB] OR ChAdOx1 nCoV-19[MeSH] OR "Oxford-AstraZeneca COVID Vaccine*"[TIAB] OR "ChAdOx1 COVID-19 Vaccine*"[TIAB] OR AZD1222[TIAB] OR Covishield[TIAB] OR 2019-nCoV Vaccine mRNA-1273[MeSH] OR "2019 nCoV Vaccine mRNA 1273*"[TIAB] OR "COVID-19 Vaccine Moderna*"[TIAB] OR mRNA-1273*[TIAB] OR "mRNA-1273.211*"[TIAB] OR Spikevax*[TIAB] OR Moderna COVID-19 Vaccine, Bivalent[MeSH]): 91,362 results

#2: (Cognitive Dysfunction[MeSH] OR "Cognitive Dysfunction*"[TIAB] OR "Cognitive Disorder*"[TIAB] OR "Cognitive Impairment*"[TIAB] OR "Mild Cognitive Impairment*"[TIAB] OR "Cognitive Decline*"[TIAB] OR Dementia[MeSH] OR "Dementia*"[TIAB] OR Alzheimer Disease[MeSH] OR "Alzheimer Disease*"[TIAB] OR Dementia, Vascular[MeSH] OR "Vascular Dementia*"[TIAB]): 436,223 results

#3: #1 AND #2: 257 results

Scopus (n= 927)

#1: TITLE-ABS-KEY(("Influenza Vaccine*" OR "Influenza Virus Vaccine*" OR "Flu Vaccine*" OR "Trivalent Live Attenuated Influenza Vaccine" OR "LAIV Vaccine*" OR "Universal Influenza Vaccine*" OR "Universal Flu Vaccine*" OR "High Dose Trivalent Influenza Vaccine*" OR "Quadrivalent Influenza Vaccine*" OR "Trivalent Influenza Vaccine*" OR "Monovalent Influenza Vaccine*" OR "Respiratory Syncytial Virus Vaccine*" OR "RSV Vaccine*" OR "Pneumococcal Vaccine*" OR "Pnu Imune Vaccine*" OR "Pneumococcal Polysaccharide Vaccine*" OR "Pneumovax" OR "COVID-19 Vaccine*" OR "SARS2 Vaccine*" OR "Coronavirus Disease 2019 Vaccine*" OR "2019-nCoV Vaccine*" OR "SARS-CoV-2 Vaccine*" OR "COVID-19 Virus Vaccine*" OR "BNT162 Vaccine*" OR "COVID-19 Vaccine Pfizer-BioNTech*" OR "Tozinameran" OR "BNT-162B2*" OR "Comirnaty" OR "Pfizer Covid-19 Vaccine*" OR "Oxford-AstraZeneca COVID Vaccine*" OR "ChAdOx1 COVID-19 Vaccine*" OR "AZD1222" OR "Covishield" OR "2019 nCoV Vaccine mRNA 1273*" OR "COVID-19 Vaccine Moderna*" OR mRNA-1273* OR "mRNA-1273.211*")): 132,158 results

#2: TITLE-ABS-KEY(("Cognitive Dysfunction*" OR "Cognitive Disorder*" OR "Cognitive Impairment*" OR "Mild Cognitive Impairment*" OR "Cognitive Decline*" OR "Dementia*" OR "Alzheimer Disease*" OR "Vascular Dementia*")): 639,915 results

#3: #1 AND #2: 927 results

Web of Science (n= 158)

#1: (TS=("Influenza Vaccine*" OR "Influenza Virus Vaccine*" OR "Flu Vaccine*" OR "Trivalent Live Attenuated Influenza Vaccine" OR "LAIV Vaccine*" OR "Universal Influenza Vaccine*" OR "Universal Flu Vaccine*" OR "High Dose Trivalent Influenza Vaccine*" OR "Quadrivalent Influenza Vaccine*" OR "Trivalent Influenza Vaccine*" OR "Monovalent Influenza Vaccine*" OR "Respiratory Syncytial Virus Vaccine*" OR "RSV Vaccine*" OR "Pneumococcal Vaccine*" OR "Pnu Imune Vaccine*" OR "Pneumococcal Polysaccharide Vaccine*" OR "Pneumovax" OR "COVID-19 Vaccine*" OR "SARS2 Vaccine*" OR "Coronavirus Disease 2019 Vaccine*" OR "2019-nCoV Vaccine*" OR "SARS-CoV-2 Vaccine*" OR "COVID-19 Virus Vaccine*" OR "BNT162 Vaccine*" OR "COVID-19 Vaccine Pfizer-BioNTech*" OR "Tozinameran" OR "BNT-162B2*" OR "Comirnaty" OR "Pfizer Covid-19 Vaccine*" OR "Oxford-AstraZeneca COVID Vaccine*" OR "ChAdOx1 COVID-19 Vaccine*" OR "AZD1222" OR "Covishield" OR "2019 nCoV Vaccine mRNA 1273*" OR "COVID-19 Vaccine Moderna*" OR mRNA-1273* OR "mRNA-1273.211*")): 59,424 results

#2: (TS=("Cognitive Dysfunction*" OR "Cognitive Disorder*" OR "Cognitive Impairment*" OR "Mild Cognitive Impairment*" OR "Cognitive Decline*" OR "Dementia*" OR "Alzheimer Disease*" OR "Vascular Dementia*")): 377,601 results

#3: #1 AND #2: 158 results
